# Supplementary material for: On the damage tolerance of 3-D printed Mg-Ti interpenetrating-phase composites with bioinspired architectures
Source: Nat Commun. 2022 Jun 6;13:3247. doi: 10.1038/s41467-022-30873-9 (PMC9170714; doi:10.1038/s41467-022-30873-9)
Supplement: Supplementary file 1 — Supplementary Information [file 41467_2022_30873_MOESM1_ESM.pdf]

On the damage tolerance of 3-D printed Mg-Ti interpenetrating-phase composites with bioinspired architectures

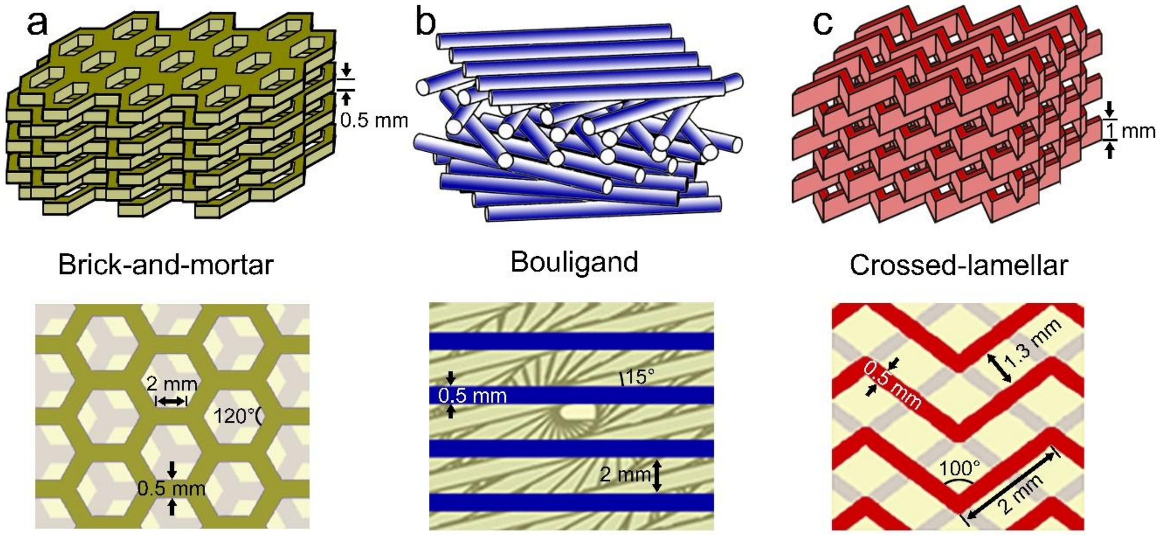

Supplementary Figure 1. Detailed dimensions and orientation characteristics of the 3-D models for bioinspired architectures. These models were used to guide the 3-D printing of Ti-6Al-4V scaffolds with bioinspired **a** brick-and-mortar, **b** Bouligand, and **c** crossed-lamellar architectures.

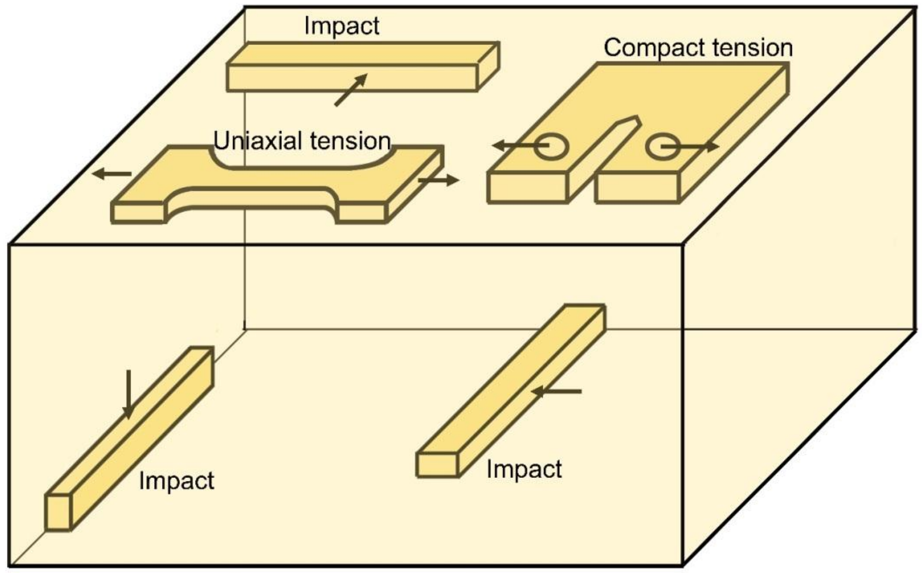

Supplementary Figure 2. Schematic illustration of the loading configurations of samples for mechanical testing for the bioinspired Mg-Ti composites. The spatial arrangements of constituents are fully in line with those shown in Supplementary Figure 1 for the brick-and-mortar, Bouligand and crossed-lamellar architectures.

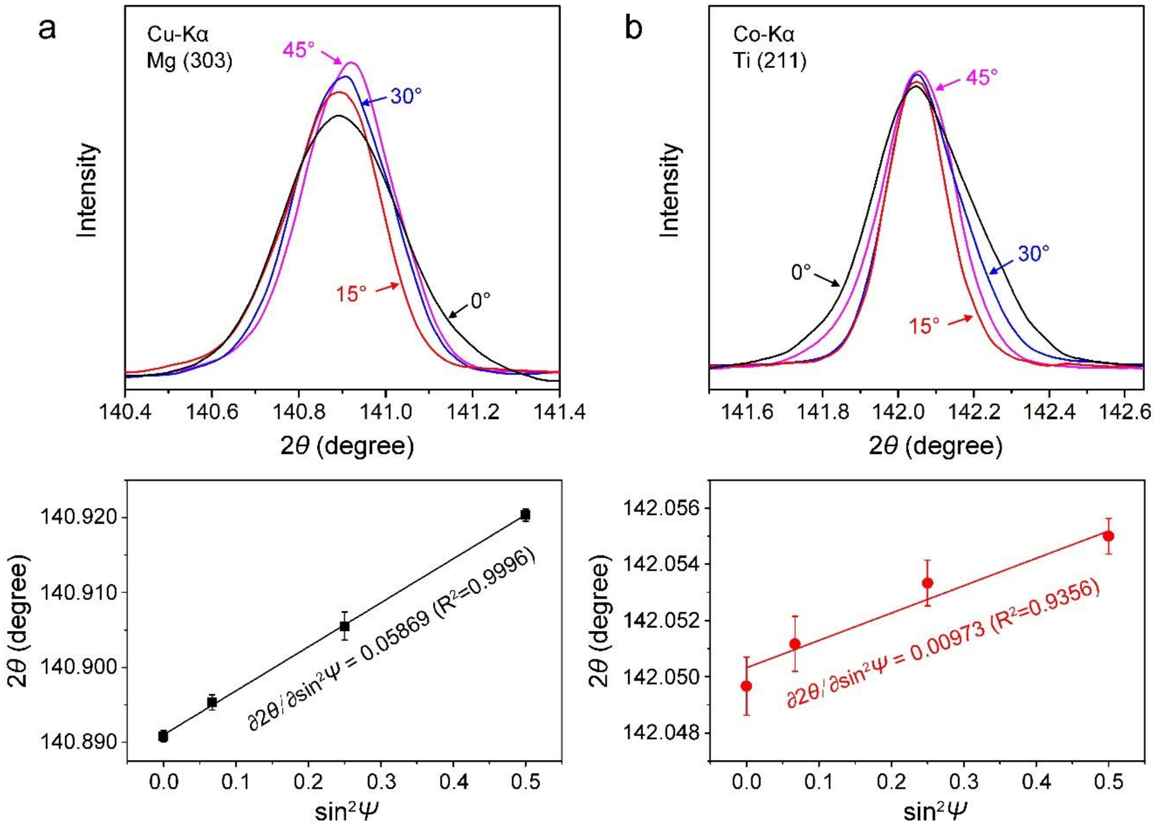

Supplementary Figure 3. Residual stress measurements for the bioinspired Mg-Ti composites. X-ray diffraction peaks at incidence angles Ψ of 0 °, 15 °, 30 ° and 45 ° and the linear fitting results of $\partial 2\theta/\partial \sin^2\Psi$ in the 2θ-$\sin^2\Psi$ plots for **a** Mg (303) plane under Cu-Kα radiation and **b** Ti (211) plane under Co-Kα radiation. θ is the X-ray diffraction angle. Error bars represent standard deviation. The crossed-lamellar architecture was taken as an example here. The residual stresses were measured to be approximately -5.4 MPa and -2.4 MPa, respectively for Mg (303) plane and Ti (211) plane in the composite. These values are lower than the strengths of the two phases by one to two orders of magnitude.

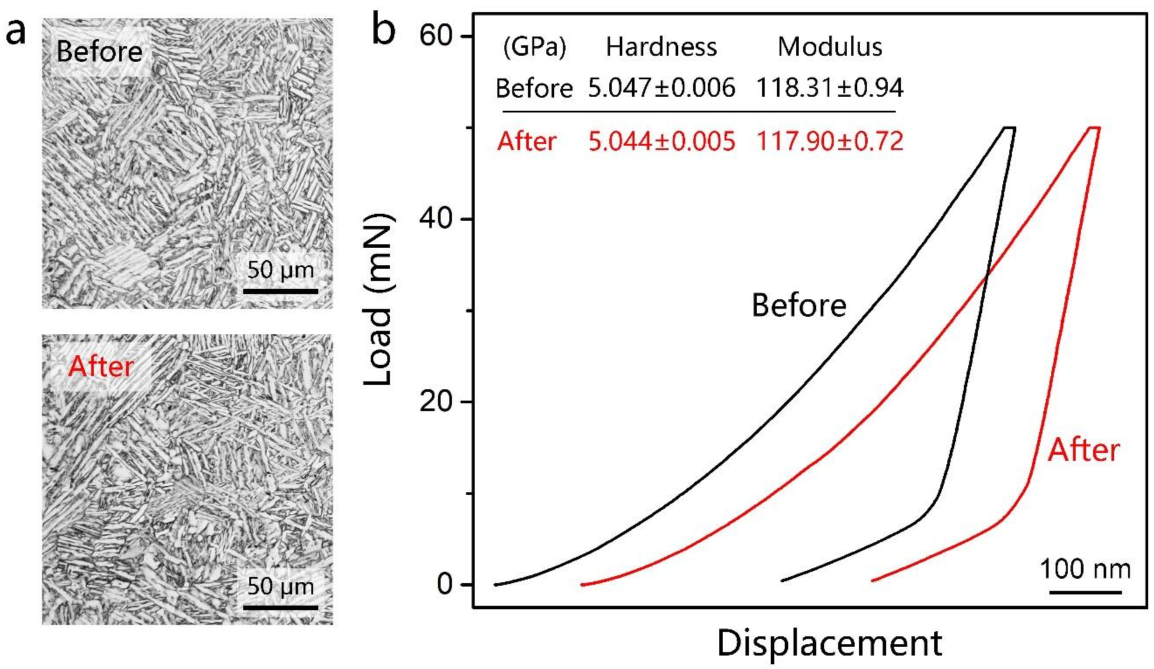

Supplementary Figure 4. Microstructures and mechanical properties of the Ti-6Al-4V scaffold before and after melt infiltration. a Representative microstructures and **b** load-displacement curves measured by nanoindentation testing along with the deduced hardness and elastic modulus (the curve for the infiltrated sample was horizontally shifted for clarity). The nanoindentation tests were conducted using an Agilent G200 nanoindenter with a diamond Berkovich tip. The loading and unloading rates were set to be 1000 mN/min with a holding time of 10 s at 50 mN. The scaffold exhibited similar microstructures and mechanical properties before and after melt infiltration.

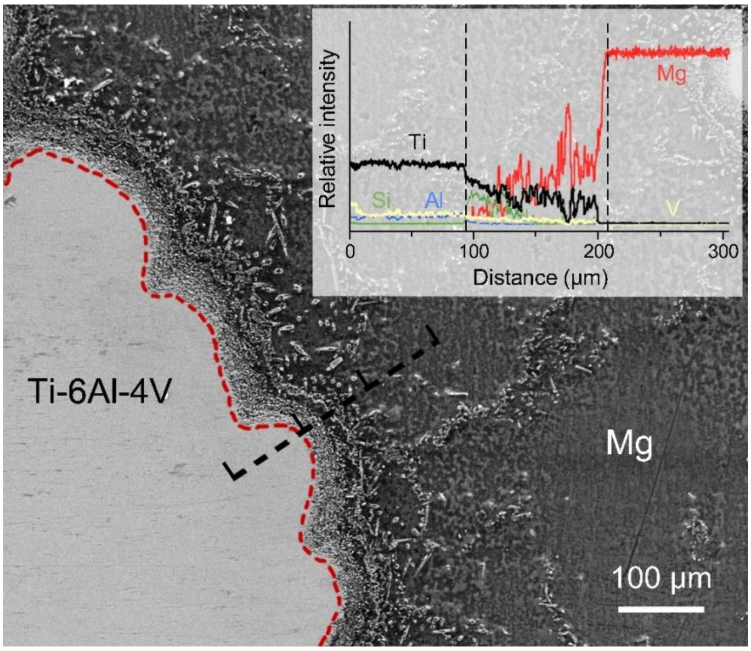

Supplementary Figure 5. Microstructural and chemical characteristics of the interface between Ti-6Al-4V and Mg phases in the infiltrated composites. The Ti-6Al-4V and Mg phases demonstrate a rough interface (as indicated by the dashed red curve) and show materials mixing over a range of ~100 μm, as evidenced by the gradual changes in the contents of Mg and Ti elements. The inset shows the energy dispersive X-ray spectroscopy results about the linear distributions of elements across the interface (along the dashed black line).

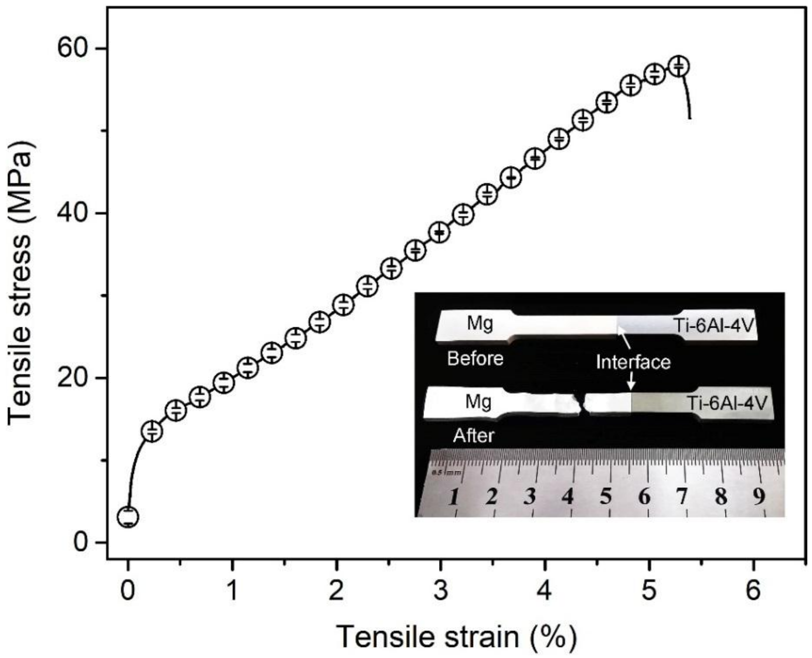

Supplementary Figure 6. Tensile stress-strain curve of the combination of Ti-6Al-4V and Mg phases bonded by a straight interface at the gauge section. The ultimate tensile strength of the combination was measured to be around 58 MPa, approximating that of pure Mg. The inset shows the overall appearances of samples before and after tensile testing. The fracture occurred in the Mg phase rather than along the interface, indicating a strong interfacial bonding with the interfacial strength higher than that of Mg.

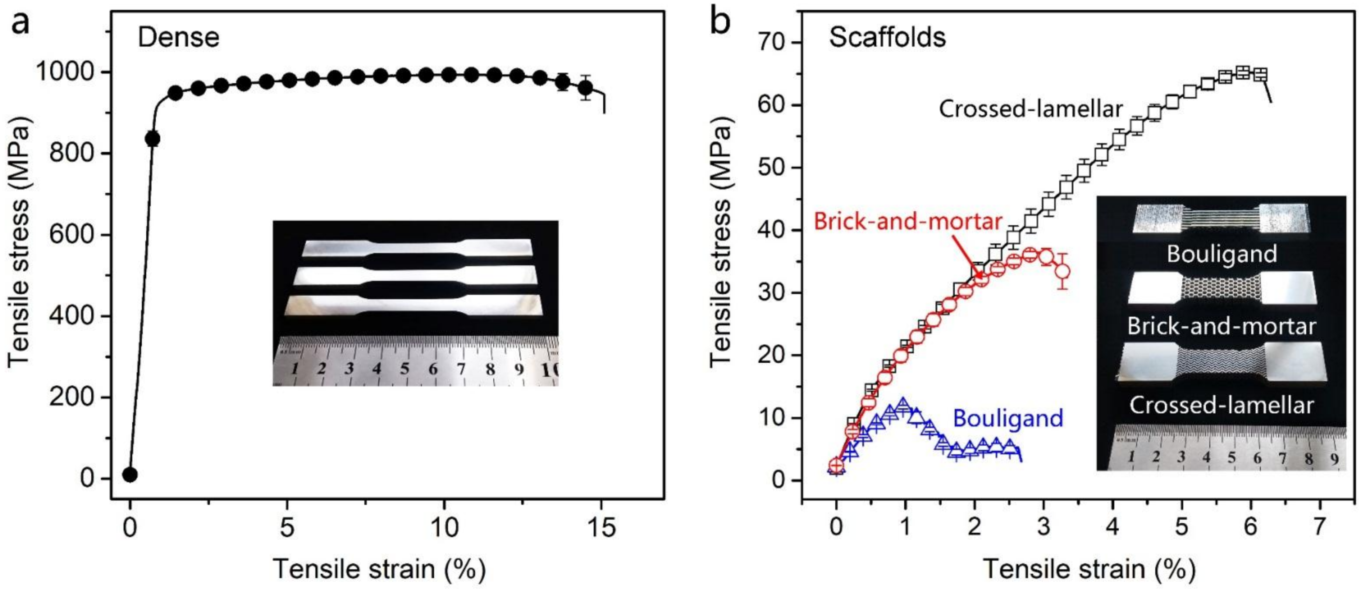

Supplementary Figure 7. Tensile properties of the 3-D printed dense Ti-6Al-4V alloy and Ti-6Al-4V scaffolds with different architectures. Tensile stress-strain curves of the 3-D printed **a** dense Ti-6Al-4V alloy and **b** Ti-6Al-4V scaffolds with bioinspired brick-and-mortar, Bouligand, and crossed-lamellar architectures without infiltration of Mg. The insets show the dog-bone shaped tensile specimens of them.

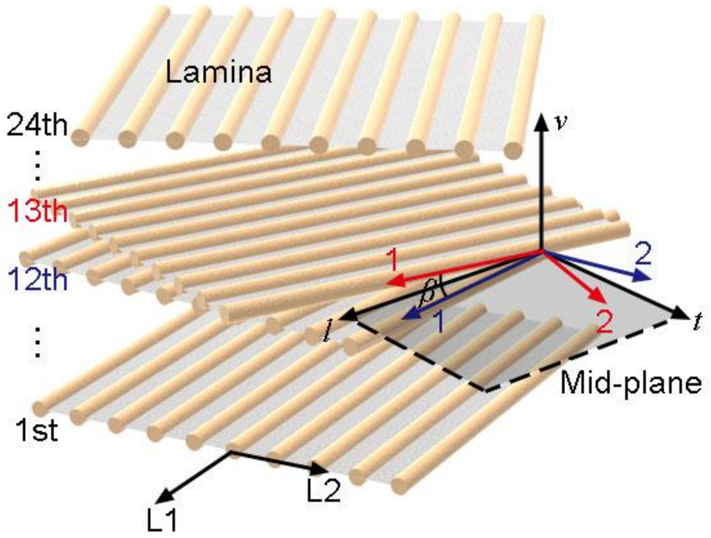

Supplementary Figure 8. Schematic illustration of the coordinate systems for the bioinspired Bouligand architecture. The architecture contains a total of 24 laminae with a twisting angle of β between fibers in adjacent laminae. L1 and L2 axes of the system are respectively parallel and perpendicular to the axes of fibers in the 1st lamina.1 and 2 axes for each lamina are respectively parallel and perpendicular to the axes of fibers in the lamina. *l* and *t* axes are respectively parallel and perpendicular to the bisector of inclination angle between the 1 axes of the 12th and 13th laminae. *v* axis is normal to these laminae.

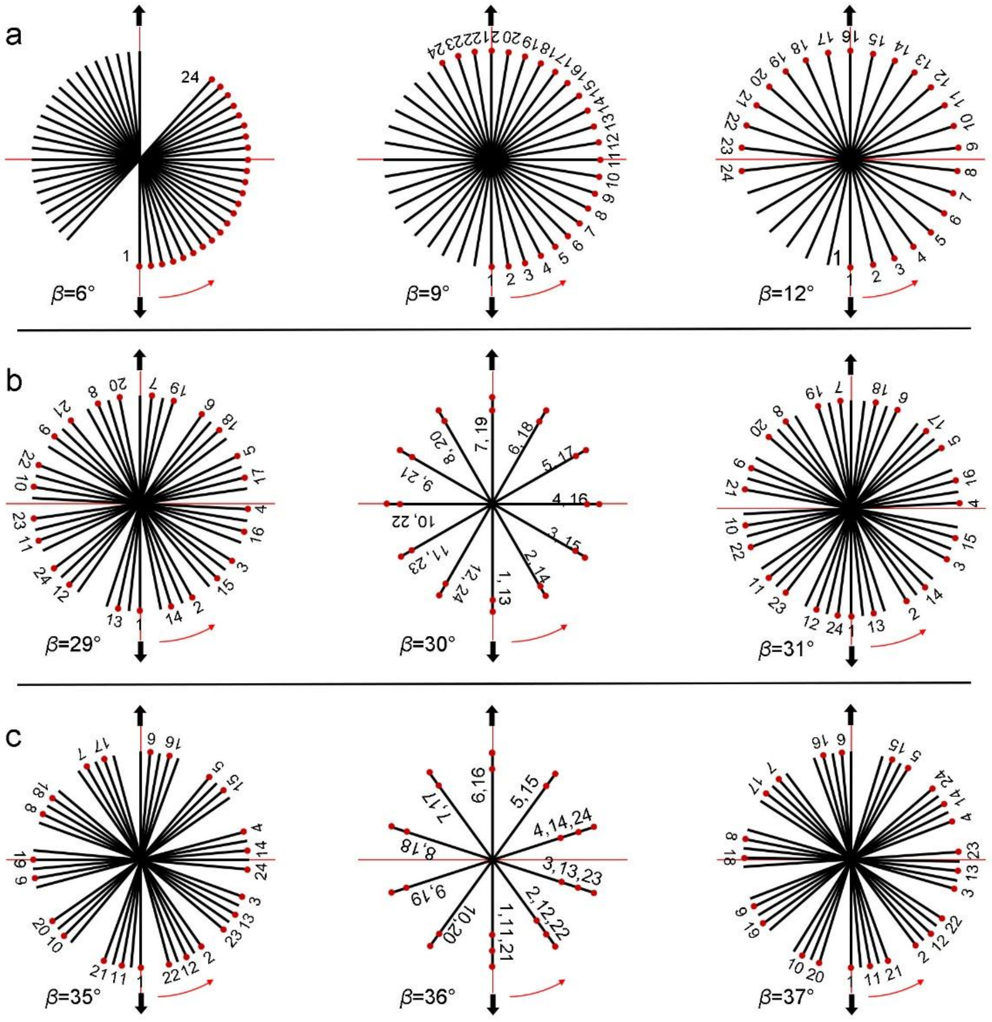

Supplementary Figure 9. Schematic illustrations of the orientations of fibers for the Bouligand architecture. The twisting angles between laminae are respectively **a** β=6°, 9°, and 12°, **b** 29°, 30°, and 31°, and **c** 35°, 36°, and 37°. The fiber orientations in different laminae, as indicated by the serial numbers, are represented by the black lines. The black and red arrows indicate the loading direction and twisting direction of laminae, respectively. **a** As β increases from 6° to 9° and then to 12°, the overall deviation of fibers in the laminate firstly increases and then decreases, leading a wavy behavior in the modulus/strength diagrams for the Bouligand architecture. **b** At specific angles that are divisible by 90° (such as β=30°), the fibers in some of the layers become perpendicular to the loading direction and thereby play a minimal role in stiffening/strengthening the composite, leading to the minimum values on the diagrams. **c** At specific angles especially those are divisible by 180° but not divisible by 90° (such as β=36°), the fibers at some of the layers are aligned right along the loading direction and none of them are perpendicular to the loading direction, leading to the peak points on the diagrams.

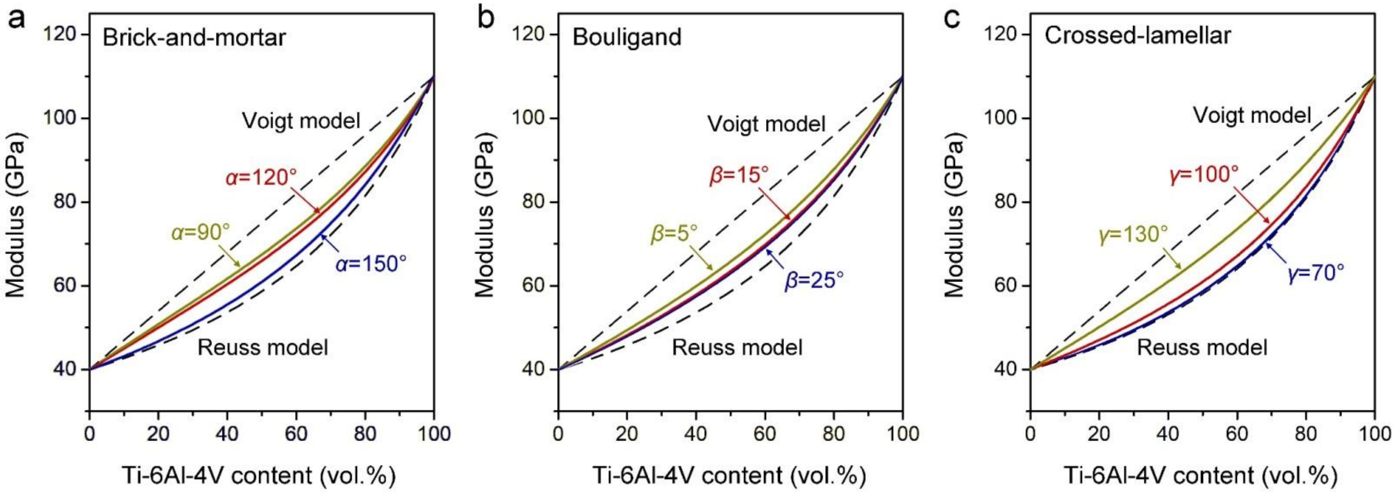

Supplementary Figure 10. Dependences of the Young's modulus on the volume fraction of Ti-6Al-4V phase in the composites. Theoretical results for the variations in the Young's modulus as a function of the volume fraction of Ti-6Al-4V phase in the composites with bioinspired **a** brick-and-mortar, **b** Bouligand, and **c** crossed-lamellar architectures at fixed values of specific angles α, β and γ. The dashed curves indicate the upper and lower bounds determined respectively by the Voigt and Reuss models in line with the rule-of-mixtures.

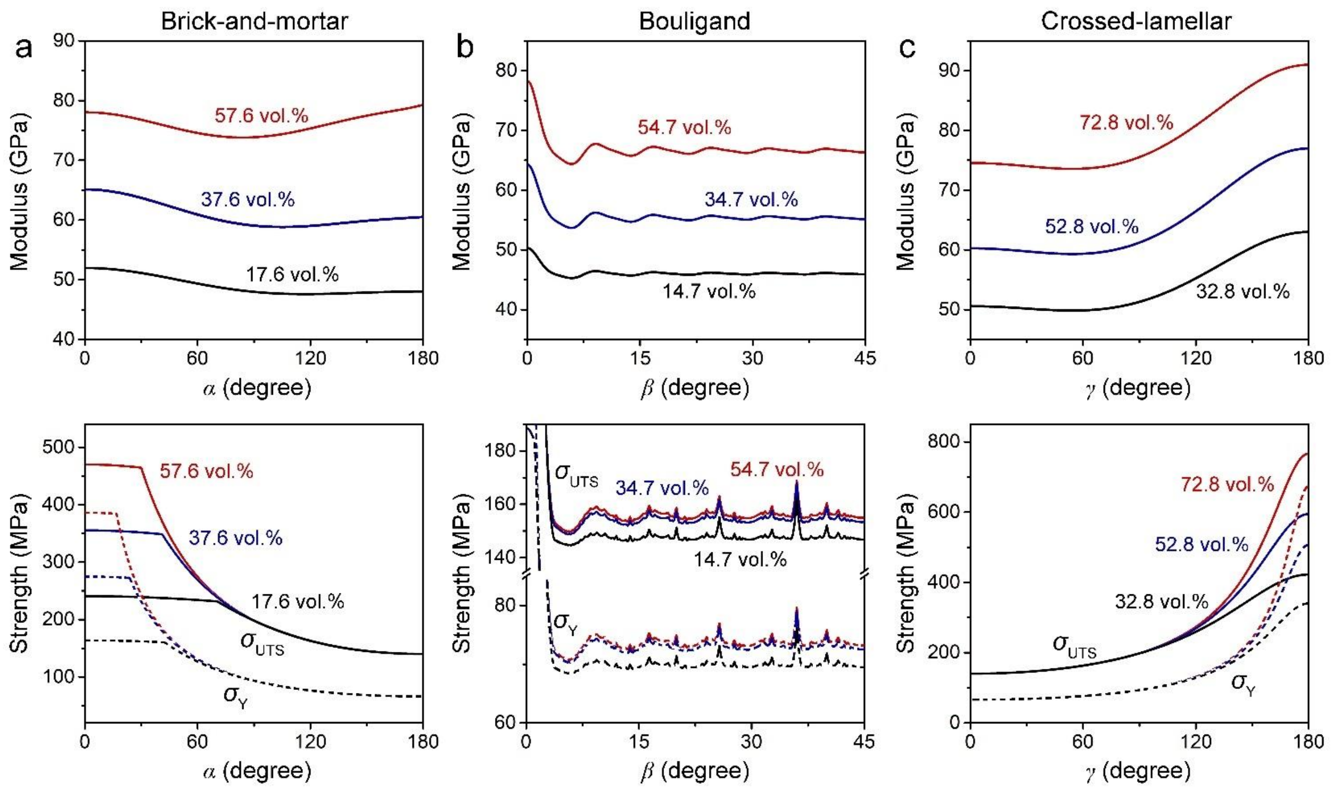

Supplementary Figure 11. Dependences of tensile properties on the specific angles in the composites. Theoretical results for the variations in the Young's modulus, yield strength σ_{YS} and ultimate tensile strength σ_{UTS} as a function of the specific angles α, β and γ, for the composites with bioinspired **a** brick-and-mortar, **b** Bouligand, and **c** crossed-lamellar architectures at fixed volume fractions of Ti-6Al-4V phase. Three different volume fractions of Ti-6Al-4V phase with 20 vol.% addition and subtraction on the basis of experimental data were considered for each type of architecture. The strengths of the brick-and-mortar architecture are comparable for different phase constitutions at the relatively large range of α because they are mainly dominated by the weak Mg phase (failure is prone to occur in element II). Similar case occurs for the cross-lamellar architecture at the relatively small range of γ.

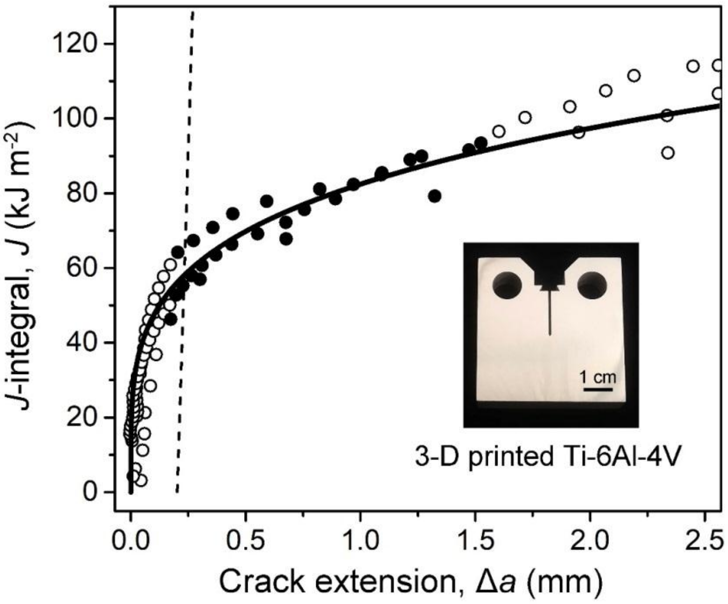

Supplementary Figure 12. Fracture toughness of 3-D printed dense Ti-6Al-4V alloy. The variation in the *J*-integral as a function of the crack extension Δa is manifested by the fitted R-curve for the 3-D printed dense Ti-6Al-4V alloy. The dashed line for determining the fracture toughness has a slope of $(\sigma_{\mathrm{YS}} + \sigma_{\mathrm{UTS}})$ and corresponds to a 0.2 mm offset strain. The inset shows the sample for compact tension testing.

Supplementary Note 1. Young's modulus of the structural element

Under plane-strain conditions, the elastic constants of the basic structural element where the reinforcements are unidirectionally aligned can be accessed from those of the constituents according to the rule-of-mixtures. Specifically, the Young's moduli along the 1 and 2 axes, E_1 and E_2, can be obtained in line with, respectively, the Voigt and Reuss models (because the stresses and strains are identical between constituents respectively in these configurations) as [1,2]:

$$E_1 = E_{\mathrm{Ti}} V_{\mathrm{Ti}} + E_{\mathrm{Mg}} V_{\mathrm{Mg}}, \tag{1}$$

$$E_2 = \frac{E_{\mathrm{Ti}} E_{\mathrm{Mg}}}{V_{\mathrm{Ti}} E_{\mathrm{Mg}} + V_{\mathrm{Mg}} E_{\mathrm{Ti}}}. \tag{2}$$

The subscripts Ti and Mg represent the constituents of Ti-6Al-4V and pure Mg in the composites. $\mathrm{V_{Ti}}$ and $\mathrm{V_{Mg}}$ are the volume fractions of the constituents with $\mathrm{V_{Ti}} + \mathrm{V_{Mg}} = 1$. The shear moduli along the 1-2 and 2-1 directions, G_{12} and G_{21}, can be assessed based on the Reuss model as [1,2]:

$$G_{12} = G_{21} = \frac{G_{\mathrm{Ti}} G_{\mathrm{Mg}}}{V_{\mathrm{Ti}} G_{\mathrm{Mg}} + V_{\mathrm{Mg}} G_{\mathrm{Ti}}}. \tag{3}$$

Poisson's ratio of the element along the 1-2 direction, v_{12}, can be obtained based on the Voigt model as:

$$v_{12} = v_{\mathrm{Ti}} V_{\mathrm{Ti}} + v_{\mathrm{Mg}} V_{\mathrm{Mg}}. \tag{4}$$

The corresponding Poisson's ratio along the 2-1 direction v_{21} can then be obtained following the relationship $v_{21} = v_{12} \frac{E_1}{E_2}$.

The stiffness matrix of the element in the local coordinate system (1, 2), $\mathbf{Q}_{1,2}$, can be described using the above elastic constants as [2,3]:

$$\mathbf{Q}_{1,2} = \begin{bmatrix} \frac{E_1}{\Delta} & \frac{v_{12} E_2}{\Delta} & 0 \\ \frac{v_{21} E_1}{\Delta} & \frac{E_2}{\Delta} & 0 \\ 0 & 0 & G_{12} \end{bmatrix}, \tag{5}$$

where $\Delta = 1 - v_{12}v_{21}$. Then, the stiffness matrix of the element in the global coordinate system (L1, L2), $\mathbf{Q}$, can be obtained by transforming $\mathbf{Q}_{1,2}$ by taking into account the inclination angle between the local and global coordinate systems θ as [2,4]:

$$\mathbf{Q} = \mathbf{T}^{-1}\mathbf{Q}_{1,2}\mathbf{T}^{-\mathrm{T}}, \tag{6}$$

with

$$\mathbf{T} = \begin{bmatrix} \cos^2\theta & \sin^2\theta & 2\sin\theta\cos\theta \\ \sin^2\theta & \cos^2\theta & -2\sin\theta\cos\theta \\ -\sin\theta\cos\theta & \sin\theta\cos\theta & \cos^2\theta - \sin^2\theta \end{bmatrix}. \tag{7}$$

The compliance matrix in the (L1, L2) coordinate system, $\mathbf{S}$, can be obtained by inversing $\mathbf{Q}$ as $\mathbf{S} = \mathbf{Q}^{-1}$. On this basis, the Young's moduli along the L1 and L2 axes, E_{L1} and E_{L2}, in the (L1, L2) coordinate system can be obtained from the compliance matrix as:

$$\mathbf{S} = \begin{bmatrix} \frac{1}{E_{\mathrm{L1}}} & S_{12} & 0 \\ S_{21} & \frac{1}{E_{\mathrm{L2}}} & 0 \\ 0 & 0 & \frac{1}{G_{\mathrm{L1L2}}} \end{bmatrix}. \tag{8}$$

Supplementary Note 2. Young's modulus and strengths for the bioinspired architectures

Brick-and-mortar architecture

For the brick-and-mortar architecture, each individual lamina can be seen as a combination of two types of structural units, I and II, which are alternately arranged and serially connected along the loading direction (Figure 4b). As such, the Young's modulus of the lamina can be assessed from those of the units according to the Reuss model considering that the stresses are identical between them [1-3]. Because the spatial orientations of constituents are equivalent with respect to the loading direction for all the laminae in the entire laminate, the Young's modulus of the laminate along the loading direction L1 can be approximated using that of the individual lamina as:

$$E_{\mathrm{BAM}} = \frac{E_{\mathrm{L1,I}} E_{\mathrm{L1,II}}}{V_{\mathrm{II}} E_{\mathrm{L1,I}} + V_{\mathrm{I}} E_{\mathrm{L1,II}}}, \tag{9}$$

where $E_{\mathrm{L1,I}}$ and $E_{\mathrm{L1,II}}$ are the Young's moduli of the units I and II. V_{I} and V_{II} are the volume fractions of units I and II in the lamina and can be described using the apex angle of the hexagonal bricks α as $V_{\mathrm{I}} = 1/(1+\cos\frac{\alpha}{2})$ and $V_{\mathrm{II}} = \cos\frac{\alpha}{2}/(1+\cos\frac{\alpha}{2})$, respectively. Therefore, the Young's modulus of the laminate with brick-and-mortar architecture can be described as a function of the specific angle α as:

$$E_{\mathrm{BAM}} = (1+\cos\frac{\alpha}{2}) \frac{E_{\mathrm{L1,I}} E_{\mathrm{L1,II}}}{E_{\mathrm{L1,I}} \cos\frac{\alpha}{2} + E_{\mathrm{L1,II}}}. \tag{10}$$

$E_{\mathrm{L1,I}}$ and $E_{\mathrm{L1,II}}$ can be obtained by transforming the stiffness matrices of the units in the local coordinate system (1, 2) to the global one (L1, L2), as detailed in Supplementary Note 1. Specifically, for the unit II, the neighboring edges of bricks are systematically inclined by an identical angle of $\pm\alpha/2$ with respect to the loading direction. As such, these edges can be considered to be equivalent in terms of their effects on the Young's modulus and strengths of the lamina. The inclination angles between (L1, L2) and (1, 2)

coordinate systems for the transformation of stiffness matrices are $0°$ and $\alpha/2$, respectively, for units I and II. The volume fractions of the Ti-6Al-4V phase in units I and II can be described using that of the entire laminate V_{Ti} as $(1+\cos\frac{\alpha}{2})V_{\mathrm{Ti}}/3$ and $(2+2\cos^{-1}\frac{\alpha}{2})V_{\mathrm{Ti}}/3$, respectively.

Bouligand architecture

A laminate consisting of a total of 24 laminae through its thickness direction is considered for formulating the Young's modulus of the Bouligand architecture. As illustrated in Supplementary Figure 8, these laminae having a thickness of t_0 are twisted in a clockwise fashion to form an antisymmetric conformation, *i.e.*, the upper and lower laminae at an equal *v*-distance from the mid-plane exhibit opposite rotations. The twisting angle between adjacent laminae is denoted as β. In this scenario, the rotation angle of the kth lamina with respect to the coordinate system of the mid-plane, which is described using that of the local coordinate systems of the 12th and 13th laminae (Supplementary Figure 8), can be described as:

$$\omega_k = (k - 12.5)\beta. \tag{11}$$

The distance of the kth lamina from the mid-plane can be described as:

$$v_k = (k - 12)t_0. \tag{12}$$

Then, the principal forces $\boldsymbol{\sigma}$ and moments $\mathbf{m}$ of the laminate, both normalized by the area, can be correlated to the in-plane strains $\boldsymbol{\varepsilon}_0$ and curvatures $\boldsymbol{\kappa}$ following the relationship [2,4]:

$$\begin{bmatrix}\boldsymbol{\sigma}\\ \mathbf{m}\end{bmatrix} = \begin{bmatrix}\mathbf{A} & \mathbf{B}\\ \mathbf{B} & \mathbf{D}\end{bmatrix}\begin{bmatrix}\boldsymbol{\varepsilon}_0\\ \boldsymbol{\kappa}\end{bmatrix}. \tag{13}$$

Vectors are used here for all the parameters to encompass the different components along different directions. $\mathbf{A}$, $\mathbf{B}$ and $\mathbf{D}$ are the stiffness matrices for the stress states of extension, extension-bending coupling and bending, which are given by [2,4]:

$$\mathbf{A} = \frac{1}{2nt_0}\sum_{k=1}^{2n}[\mathbf{Q}_k(v_k - v_{k-1})],$$

$$\mathbf{B} = \frac{1}{4nt_0}\sum_{k=1}^{2n}[\mathbf{Q}_k({v_k}^2 - {v_{k-1}}^2)],$$

$$\mathbf{D} = \frac{1}{6nt_0}\sum_{k=1}^{2n}[\mathbf{Q}_k({v_k}^3 - {v_{k-1}}^3)]. \tag{14}$$

n is half the total number of laminae in the laminate which equals 12. $\mathbf{Q}_k$ is the stiffness matrix of the kth lamina which can be obtained by transforming its stiffness matrix in the local coordinate system (1, 2) to that of the mid-plane (*l*, *t*) using the rotation angle ω_k, as detailed in Supplementary Note 1.

The above relationship of Eq. (10) can then be converted to:

$$\begin{bmatrix}\boldsymbol{\varepsilon}_0\\ \boldsymbol{\kappa}\end{bmatrix} = \begin{bmatrix}\mathbf{a} & \mathbf{b}\\ \mathbf{c} & \mathbf{d}\end{bmatrix}\begin{bmatrix}\boldsymbol{\sigma}\\ \mathbf{m}\end{bmatrix}, \tag{15}$$

where $\begin{bmatrix}\mathbf{a} & \mathbf{b}\\ \mathbf{c} & \mathbf{d}\end{bmatrix}$ is the compliance matrix for the entire laminate with $\begin{bmatrix}\mathbf{a} & \mathbf{b}\\ \mathbf{c} & \mathbf{d}\end{bmatrix} = \begin{bmatrix}\mathbf{A} & \mathbf{B}\\ \mathbf{B} & \mathbf{D}\end{bmatrix}^{-1}$. When the laminate is subject to in-plane stress, *i.e.*, with $\boldsymbol{\sigma} \neq \mathbf{0}$ and $\mathbf{m} = \mathbf{0}$, the plane-strain vector of the laminate can be simplified as:

$$\boldsymbol{\varepsilon}_0 = \mathbf{a}\boldsymbol{\sigma}. \tag{16}$$

The compliance matrix of the laminate in the global coordinate system (L1, L2), $\mathbf{s}$, can be obtained by transforming the compliance matrix in the mid-plane coordinate system (*l*, *t*), $\mathbf{a}$, by taking into account their inclination angle $\Phi = (n - \frac{1}{2})\beta$, as [4]:

$$\mathbf{s} = \mathbf{t}^{\mathrm{T}}\mathbf{a}\mathbf{t}, \tag{17}$$

with

$$\mathbf{t} = \begin{bmatrix}\cos^2\Phi & \sin^2\Phi & 2\sin\Phi\cos\Phi\\ \sin^2\Phi & \cos^2\Phi & -2\sin\Phi\cos\Phi\\ -\sin\Phi\cos\Phi & \sin\Phi\cos\Phi & \cos^2\Phi - \sin^2\Phi\end{bmatrix}. \tag{18}$$

The Young's modulus of the entire laminate with Bouligand architecture can then be obtained from the component of $\mathbf{s}$ as:

$$E_{\mathrm{B}} = \frac{1}{s_{11}}. \tag{19}$$

Crossed-lamellar architecture

For the crossed-lamellar architecture, each individual lamina of the laminate can be seen as a combination of two types of structural units, i and ii, which are alternately arranged and serially connected along the loading direction, similar to the case for the brick-and-mortar architecture. The reinforcement platelets are inclined by opposite angles of $\pm\gamma/2$ with respect to the loading direction in the units i and ii. The Young's modulus of the entire laminate, E_{CL}, can be approximated using that of the individual lamina as the orientations of constituents with respect to the loading direction are essentially equivalent in all the laminae in the laminate.

The Young's modulus of the lamina along the loading direction L1 can be obtained according to the rule-of-mixtures based on the Reuss model as [1,2]:

$$E_{\mathrm{CL}} = \frac{E_{\mathrm{L1,i}}E_{\mathrm{L1,ii}}}{V_{\mathrm{ii}}E_{\mathrm{L1,i}}+V_{\mathrm{i}}E_{\mathrm{L1,ii}}} = \frac{2E_{\mathrm{L1,i}}E_{\mathrm{L1,ii}}}{E_{\mathrm{L1,i}}+E_{\mathrm{L1,ii}}}, \tag{20}$$

where $E_{\mathrm{L1,i}}$ and $E_{\mathrm{L1,ii}}$ are the Young's moduli of the units i and ii. $E_{\mathrm{L1,i}}$ and $E_{\mathrm{L1,ii}}$ can be formulated using those of the constituents as a function of their inclination angles with respect to the loading direction, as detailed in Supplementary Note 1. V_{i} and V_{ii} are the volume fractions of the units i and ii in the lamina and are both equal to 0.5. The inclination angles between the local and global coordinate systems for the units i and ii are 90°-$\gamma/2$ and $\gamma/2$-90°, respectively. The volume fractions of the Ti-6Al-4V and Mg phases in the units i and ii are equal to that in the entire laminate.

Supplementary Note 3. Failure stress of the structural element

The Tsai-Hill failure criterion has proven to be applicable to a range of composites, including those with bioinspired architectures [3-8]. The critical stresses causing the failure (either yielding or fracture under tension) of the structural element can be determined by such a criterion following the relationship [5-7]:

$$\frac{{\sigma_1}^2}{X^2} - \frac{\sigma_1\sigma_2}{X^2} + \frac{{\sigma_2}^2}{Y^2} + \frac{{\tau_{12}}^2}{S^2} = 1, \qquad (21)$$

where σ_1 and σ_2 are the principal stresses along the axes 1 and 2, and τ_{12} is the shear stress along the 1-2 direction in the local coordinate system (1, 2). X and Y represent the critical tensile failure stresses along the principal axes 1 and 2, respectively, and S is the shear failure stress along the 1-2 direction. X can be approximated to be the weighted average of the tensile failure stresses of the Ti-6Al-4V and Mg phases by their volume fractions. Y and S can be approximated using those of the coarse-grained pure Mg considering that the failure of the composite in these loading configurations is principally governed by the weak matrix [4,7]. The values of Y and S were set to be identical for different architectures and were determined by fitting the experimental data.

Considering the inclination of the local coordinate system (1, 2) with respect to the global one (L1, L2), σ_1, σ_2 and τ_{12} can be described using the critical failure stress of the structural element along the loading direction, σ_f, as:

$$\sigma_1 = \sigma_\mathrm{f}\cos^2\theta,$$

$$\sigma_2 = \sigma_\mathrm{f}\sin^2\theta,$$

$$\tau_{12} = \sigma_\mathrm{f}\cos\theta\sin\theta, \qquad (22)$$

where θ is the inclination angle between the local and global coordinate systems. As such, the failure stress of the element, which represents either the yield strength or the ultimate tensile strength, can be obtained by converting Eq. (21) to the following form:

$$\frac{(\sigma_{\mathrm{f}}{}^{2}\cos^{4}\theta)}{X^{2}} + (\frac{1}{S^{2}} - \frac{1}{X^{2}})\sigma_{\mathrm{f}}{}^{2}\cos^{2}\theta\sin^{2}\theta + \frac{(\sigma_{\mathrm{f}}{}^{2}\sin^{4}\theta)}{Y^{2}} = 1. \tag{23}$$

Supplementary Note 4. Strengths of the laminate with bioinspired architectures

Brick-and-mortar architecture

For the brick-and-mortar architecture, the failure stress of the entire laminate, $\sigma_{\mathrm{f,BAM}}$, can be approximated using that of the individual lamina as the orientations of constituents with respect to the loading direction are essentially equivalent in all the laminae in the laminate. As the units I and II are serially connected in the lamina, the failure stress of the individual lamina can be determined using the lower one of the units. For the unit I, the failure stress equals to that along the 1 axis with $\sigma_{\mathrm{f,I}} = X_{\mathrm{I}}$. As the walls of bricks are inclined at $\pm\alpha/2$ with respect to the loading direction, the failure stress of unit II, $\sigma_{\mathrm{f,II}}$, can be determined according to the Tsai-Hill failure criterion by incorporating the above inclination angle into Eq. (23) as:

$$\sigma_{\mathrm{f,II}} = [\frac{\cos^4(\alpha/2)}{{X_{\mathrm{II}}}^2} + (\frac{1}{{S_{\mathrm{II}}}^2} - \frac{1}{{X_{\mathrm{II}}}^2})\sin^2(\alpha/2)\cos^2(\alpha/2) + \frac{\sin^4(\alpha/2)}{{Y_{\mathrm{II}}}^2}]^{-\frac{1}{2}}. \quad (24)$$

Therefore, the failure stress of the entire laminate can be formulated as a function of the apex angle of the bricks, α, as:

$$\sigma_{\mathrm{f,BAM}} = \min\{X_{\mathrm{I}}, [\frac{\cos^4(\alpha/2)}{{X_{\mathrm{II}}}^2} + (\frac{1}{{S_{\mathrm{II}}}^2} - \frac{1}{{X_{\mathrm{II}}}^2})\sin^2(\alpha/2)\cos^2(\alpha/2) + \frac{\sin^4(\alpha/2)}{{Y_{\mathrm{II}}}^2}]^{-\frac{1}{2}}\}, \quad (25)$$

where X_{I}, X_{II}, Y and S can be determined following the methods as detailed in Supplementary Note 3.

Bouligand architecture

For the Bouligand architecture, the plane-stress matrix of the laminate in the global coordinate system (L1, L2) can be expressed as [3,4]:

$$\boldsymbol{\sigma}_{\mathrm{L}} = \mathbf{R}\sigma_{\mathrm{f,B}}\,, \quad (26)$$

with $\mathbf{R} = [1 \quad i \quad j]^{\mathrm{T}}$. $\sigma_{\mathrm{f,B}}$ is the critical stress along the loading direction L1, and i and j are the ratios of, respectively, the stress along the L2 direction and the shear stress along

the L1-L2 direction in the global coordinate system with respect to $\sigma_{\mathrm{f,B}}$. As the laminate is free of bending or distortion deformation, the total plane-strain matrix of the laminate can be given according to the stress-strain relationship as:

$$\boldsymbol{\varepsilon}_{\mathrm{L}} = \mathbf{s}\boldsymbol{\sigma}_{\mathrm{L}} = \mathbf{sR}\sigma_{\mathrm{f,B}}, \tag{27}$$

where $\mathbf{s}$ is the compliance matrix in the global coordinate system (L1, L2). The plane strain in the kth lamina in its local coordinate system (1, 2) can be obtained by transforming $\boldsymbol{\varepsilon}_{\mathrm{L}}$ as [3,4]:

$$\boldsymbol{\varepsilon}_k = \mathbf{H}_k\boldsymbol{\varepsilon}_{\mathrm{L}} = \mathbf{H}_k\mathbf{sR}\sigma_{\mathrm{f,B}},$$

$$\mathbf{H}_k = \begin{bmatrix} \cos^2\eta_k & \sin^2\eta_k & 2\sin\eta_k\cos\eta_k \\ \sin^2\eta_k & \cos^2\eta_k & -2\sin\eta_k\cos\eta_k \\ -\sin\eta_k\cos\eta_k & \sin\eta_k\cos\eta_k & \cos^2\eta_k - \sin^2\eta_k \end{bmatrix}, \tag{28}$$

where $\eta_k = (k-1)\beta$ is the inclination angle between the local and global coordinate systems for the kth lamina. The plane stress in the kth lamina in the local coordinate system can then be given by:

$$\boldsymbol{\sigma}_k = \mathbf{Q}\boldsymbol{\varepsilon}_k = \mathbf{QH}_k\mathbf{sR}\sigma_{\mathrm{f,B}} = \mathbf{P}_k\sigma_{\mathrm{f,B}},$$

$$\mathbf{P}_k = \mathbf{QH}_k\mathbf{sR}, \tag{29}$$

where $\mathbf{Q}$ is the stiff matrix of the lamina in the local coordinate system and can be obtained in line with the methods described in Supplementary Note 1. Considering that $\boldsymbol{\sigma}_k = [\sigma_{1k} \quad \sigma_{2k} \quad \tau_{12k}]^{\mathrm{T}}$ and $\mathbf{P}_k = [P_{1k} \quad P_{2k} \quad P_{12k}]^{\mathrm{T}}$, the following relationships can be obtained for each individual lamina:

$$\sigma_{1k} = P_{1k}\sigma_{\mathrm{f,B}},$$

$$\sigma_{2k} = P_{2k}\sigma_{\mathrm{f,B}},$$

$$\tau_{12k} = P_{12k}\sigma_{\mathrm{f,B}}. \tag{30}$$

σ_{1k}, σ_{2k} and τ_{12k} are the principal stresses in the global coordinate system of the kth lamina. The critical stress for failure can then be obtained according to the Tsai-Hill failure criterion following Eq. (21) as [4-7]:

$$\sigma_{\mathrm{f,B}} = (\frac{{P_{1k}}^2 - P_{1k}P_{2k}}{{X_\mathrm{B}}^2} + \frac{{P_{2k}}^2}{{Y_\mathrm{B}}^2} + \frac{{P_{12k}}^2}{{S_\mathrm{B}}^2})^{-\frac{1}{2}}, \tag{31}$$

where X_B, Y_B and S_B can be determined following the methods as detailed in Supplementary Note 3. Accordingly, the failure stress of the laminate can be obtained by incorporating Eqs. (28)-(30) into Eq. (31).

On the above basis, the yield strength of the laminate can be accessed according to the conservative first-ply failure criterion which assumes that the laminate yields when the first lamina (*i.e.*, the lamina with the lowest strength) fails [7,8]. As such, the yield strength of the laminate can be determined using the specific failure stress corresponding to the failure of the first lamina. Similarly, the ultimate tensile strength of the laminate can be approximated using the critical stress at which all the laminae fail [4,6], *i.e.*, corresponding to the failure of the final lamina.

Crossed-lamellar architecture

For the crossed-lamellar architecture, the failure stress of the entire laminate, $\sigma_{\mathrm{f,CL}}$, can be approximated using that of the individual lamina as the orientations of constituents with respect to the loading direction are essentially equivalent in all the laminae in the laminate. Additionally, the failure stresses of the units i and ii within the individual lamina are also identical as the constituents are systematically inclined with respect to the loading direction in these units. The inclination angle can be associated with the specific γ as 90°-$\gamma/2$. As such, the failure stress of the laminate can be obtained based on the Tsai-Hill failure criterion by incorporating the inclination angle into Eq. (23) as:

$$\sigma_{\mathrm{f,CL}} = [\frac{\sin^4(\gamma/2)}{{X_\mathrm{i}}^2} + (\frac{1}{{S_\mathrm{i}}^2} - \frac{1}{{X_\mathrm{i}}^2})\sin^2(\gamma/2)\cos^2(\gamma/2) + \frac{\cos^4(\gamma/2)}{{Y_\mathrm{i}}^2}]^{-\frac{1}{2}}. \tag{32}$$

Supplementary References

[1] Reuss, A. Berechnung der fließgrenze von mischkristallen auf grund der plastizitätsbedingung für einkristalle. *Z. Angew. Math. Mech.* **9**, 49-58 (1929).

[2] Jones, R. M. *Mechanics of Composite Materials*, second ed. (CRC Press, Philadelphia, 2014).

[3] Greenfeld, I., Kellersztein, I. & Wagner, H. D. Nested helicoids in biological microstructures. *Nat. Commun.* **11**, 224 (2020).

[4] Zhang, Y., Tan, G., Zhang, M., Yu, Q., Liu, Z., Liu, Y., Zhang, J., Jiao, D., Wang, F., Zhuo, L., Zhang, Z. & Ritchie, R. O. Bioinspired tungsten-copper composites with Bouligand-type architectures mimicking fish scales. *J Mater. Sci. Technol.* **96**, 21-30 (2022).

[5] Tsai, S. W. Strength theories of filamentary structure, in fundamental aspects of fibre reinforced plastic composites, Eds: R. T. Schwartz, H. S. Schwartz (Wiley Interscience, New York, 1968).

[6] Hill, R. Theory of mechanical properties of fibre-strengthened materials: I. Elastic behaviour. *J. Mech. Phys. Solids* **12**, 199-212 (1964).

[7] Tsai, S. W. & Wu, E. M. A general theory of strength for anisotropic materials. *J. Compos. Mater.* **5**, 58-80 (1971).

[8] Dvorak, G. J. & Laws, N. Analysis of progressive matrix cracking in composite laminates II. First ply failure. *J. Compos. Mater.* **21**, 309-329 (1987).
