# Supplementary material for: On the damage tolerance of 3-D printed Mg-Ti interpenetrating-phase composites with bioinspired architectures
Source: Nat Commun. 2022 Jun 6;13:3247. doi: 10.1038/s41467-022-30873-9 (PMC9170714; doi:10.1038/s41467-022-30873-9)
Supplement: Supplementary file 3 — Description of Additional Supplementary Files [file 41467_2022_30873_MOESM3_ESM.pdf]

Description of Additional Supplementary Files

File Name: Supplementary Movie 1
Description: . X-ray computed tomographic 3-D perspective views of the cracking profiles in the quasi-static fracture toughness sample of Mg-Ti composite with bioinspired brick-and-mortar architecture.

File Name: Supplementary Movie 2
Description: X-ray computed tomographic 3-D perspective views of the cracking profiles in the quasi-static fracture toughness sample of Mg-Ti composite with bioinspired Bouligand architecture.

File Name: Supplementary Movie 3
Description: X-ray computed tomographic 3-D perspective views of the cracking profiles in the quasi-static fracture toughness sample of Mg-Ti composite with bioinspired crossed-lamellar architecture.
